# Activation of cytotoxic lymphocytes through CD6 enhances killing of cancer cells

**DOI:** 10.1007/s00262-023-03578-1

**Published:** 2024-01-27

**Authors:** Mikel Gurrea-Rubio, Qi Wu, M. Asif Amin, Pei-Suen Tsou, Phillip L. Campbell, Camila I. Amarista, Yuzo Ikari, William D. Brodie, Megan N. Mattichak, Sei Muraoka, Peggy M. Randon, Matthew E. Lind, Jeffrey H. Ruth, Yang Mao-Draayer, Shengli Ding, Xiling Shen, Laura A. Cooney, Feng Lin, David A. Fox

**Affiliations:** 1 Department of Internal Medicine, Division of Rheumatology, University of Michigan and Autoimmunity Center of Excellence, Ann Arbor, MI USA; 2 Department of Neurology, University of Michigan, Ann Arbor, MI USA; 3 Present Address: Oklahoma Medical Research Foundation, 825 NE 13th St, Oklahoma City, OK 73104 USA; 4 Xilis, Inc., Durham, NC USA; 5 Department of Immunity and Inflammation, Lerner Research Institute, Cleveland, OH USA

**Keywords:** CD6, Immunotherapy, NK, Cytotoxic lymphocyte

## Abstract

Immune checkpoint inhibitors (ICIs) have demonstrated efficacy and improved survival in a growing number of cancers. Despite their success, ICIs are associated with immune-related adverse events that can interfere with their use. Therefore, safer approaches are needed. CD6, expressed by T-lymphocytes and human NK cells, engages in cell–cell interactions by binding to its ligands CD166 (ALCAM) and CD318 (CDCP1). CD6 is a target protein for regulating immune responses and is required for the development of several mouse models of autoimmunity. Interestingly, CD6 is exclusively expressed on immune cells while CD318 is strongly expressed on most cancers. Here we demonstrate that disrupting the CD6-CD318 axis with UMCD6, an anti-CD6 monoclonal antibody, prolongs survival of mice in xenograft mouse models of human breast and prostate cancer, treated with infusions of human lymphocytes. Analysis of tumor-infiltrating immune cells showed that augmentation of lymphocyte cytotoxicity by UMCD6 is due to effects of this antibody on NK, NKT and CD8 + T cells. In particular, tumor-infiltrating cytotoxic lymphocytes from UMCD6-treated mice expressed higher levels of perforin and were found in higher proportions than those from IgG-treated mice. Moreover, RNA-seq analysis of human NK-92 cells treated with UMCD6 revealed that UMCD6 up-regulates the NKG2D-DAP10 receptor complex, important in NK cell activation, as well as its downstream target PI3K. Our results now describe the phenotypic changes that occur on immune cells upon treatment with UMCD6 and further confirm that the CD6-CD318 axis can regulate the activation state of cytotoxic lymphocytes and their positioning within the tumor microenvironment.

**Supplementary Information:**

The online version contains supplementary material available at 10.1007/s00262-023-03578-1.

## Introduction

Inhibition of immune checkpoints using monoclonal antibodies (mAbs) against CTLA-4, PD-1 and PD-L1 has revolutionized the outlook for some cancer patients. Despite their success in the clinic, the use of these immune checkpoint inhibitors can be hindered by resistance of cancer cells to lymphocyte killing and the appearance of immune-related adverse events, restricting the number of patients who achieve durable responses.

Our previous work showed that CD6, a cell surface protein expressed by T lymphocytes and human NK cells [1, 2], is essential in mouse models of multiple sclerosis [3], rheumatoid arthritis [4], and uveitis [5]. In both CD6^−/−^ mice and CD6-humanized mice treated with UMCD6, an anti-human CD6 mAb, striking reductions in clinical signs of disease, pathogenic Th1/Th17 responses and inflammatory cell infiltration into the target organs were observed. Soon after discovering CD318 (CDCP1) as the second ligand of CD6 [6], we demonstrated that interrupting the CD6-CD318 axis with a single dose of UMCD6 enhances the capacity of human PBMC (peripheral blood mononuclear cells) to kill CD318 + breast, prostate and lung cancer cells in vitro*,* and breast cancer cells *in vivo* [7]. CD318 has been extensively studied in cancer because of its correlation with higher occurrence of metastases and poor prognosis in most cancers [8], due to its role in metastasis formation through interaction with integrins and anti-apoptotic signaling via Akt [9]*.*

In light of these observations, we have evaluated the longer-term effects of interrupting the CD6-CD318 axis with UMCD6 on treatment of breast and prostate cancers in vivo and probed the mechanisms by which UMCD6 increased lymphocyte-mediated cytotoxicity against cancer cells. Weekly injections of UMCD6 increased survival and augmented killing (by PBMC or isolated NK cells) of breast and prostate cancer cells xeno-transplanted into immunodeficient mice. Next, we demonstrated that tumor-infiltrating lymphocytes (TILs) from UMCD6-treated mice contain higher proportions of cytotoxic lymphocytes and have higher cytotoxic activity capacity. We found an increased frequency of CD56^dim^/CD16^bright^ NK cells, NKT cells and CD8 + T cells, and higher perforin production in TILs from UMCD6-treated xenografted mice. These findings correlate with RNA-seq data from NK-92 cells that shows widespread changes in gene expression of several activating receptors (NKG2D-DAP10 and 2B4) and granzyme genes by UMCD6. Altogether, these results provide mechanistic support for anti-CD6 therapy as a promising mAb for cancer immunotherapy. Moreover, because UMCD6 can suppress many autoimmune syndromes by its direct effects on CD4 + T cells [3–5, 10], the use of this antibody to treat human cancer could avoid the troubling autoimmune complications frequently seen with current checkpoint inhibitors.

## Materials and methods

### Cell lines and cell culture

MDA-MB-231 (HTB-26™), a human tiple-negative (ER^−^/PR^−^/HER2^−^) breast cancer cell line, and PC-3 (CRL-1435™), a human androgen-independent prostate adenocarcinoma cell line, were used to assess immune cell killing of tumor cells in vivo. Both cell lines were purchased from the American Type Culture Collection (ATCC). MDA-MB-231 cells were cultured in RPMI-1640 medium (HyClone) supplemented with 10% heat-inactivated FBS (Biowest) and 1% antibiotic–antimycotic solution (Gibco Life Technologies). PC-3 cells were grown in Ham’s F-12 K medium (HyClone) supplemented with 10% heat-inactivated FBS and 1% antibiotic–antimycotic solution. Cell lines were maintained at 37 °C in 5% CO_2_ and detached with trypsin (HyClone) for passaging and further culture. Breast cancer line MDA-MB-468 (HTB-132™) was kindly gifted by Dr. Feng Lin from Cleveland Clinic. This cell line was also purchased from ATCC and grown in Leibovitz’s medium in the presence of 10% FBS. For the RNA sequencing experiments, we purchased NK-92 cells from ATCC. NK-92 cells were cultured in MEM-α medium (HyClone) supplemented with 12.5% FBS, 12.5% horse serum (Gibco Life Technologies), 100 IU/ml IL-2 (R&D Systems, Minneapolis, MN, USA), 0.2 mM inositol (Sigma-Aldrich, St. Louis, MO, USA), 0.02 mM folic acid (Sigma-Aldrich) and 0.1 mM mercaptoethanol (Gibco Life Technologies).

### Xenografts

All animal experiments were conducted in compliance with the Animal Care and Use Committee at the University of Michigan. To assess the efficacy of UMCD6 in vivo, female and male severe combined immunodeficient (SCID) beige mice (Charles River) were anesthetized intraperitoneally with ketamine (80–120 mg/kg) and xylazine (5–10 mg/kg) and then injected subcutaneously with either 2 × 10^6^ luciferase-infected MDA-MB-231 cells or PC-3 cells into the right flank of each mouse. When breast or prostate tumors reached volumes of at least 100 mm^3^ and were clearly visible by the In Vivo Imaging System, mice were infused with human 1 × 10^7^ PBMC or 1 × 10^6^ NK cells through the tail vein. Next, mice received intraperitoneal injections of UMCD6, anti-PD-1 or IgG control antibodies (100 µg/mouse) every 7 days. End of the study for each mouse was defined as the day on which tumors reached ≥ 2 cm in diameter and/or ulceration covered ≥ 50% of the tumor. Tumor growth was monitored by bioluminescence imaging. Briefly, mice were intraperitoneally injected with 100 µl of sterile D-luciferin at 15 mg/mL (Promega Corporation), anesthetized with isoflurane and then imaged with a Xenogen IVIS 200 bioluminescence camera. All images were normalized to the same scale and exposure time.

### Generation of MicroOrganoSpheres (MOS)

Lung tumor tissue samples were processed by Xilis Inc., and MOS were generated as described previously [11, 12]. MOS were cultured in medium containing DMEM/F12 (HyClone), HEPES (Gibco) and Glutamax (Gibco Life Technologies) and treated on day 2 with anti-PD1, UMCD6 and controls for 4 days. Annexin V green dye was used to indicate cell death and longitudinal images were taken in the Incucyte®.

### Isolation of human peripheral mononuclear cells (PBMCs) and NK cells

Freshly collected peripheral blood from healthy adults was used to isolate PBMCs by density gradient centrifugation using Ficoll-Paque (GE Healthcare) for both in vivo and in vitro experiments. Isolated PBMCs were either infused into breast cancer and prostate cancer xenografts or enriched for specific subpopulations such as NK cells. Specifically, NK cells were isolated using the EasySep® Human NK Cell Isolation Kit (STEMCELL Technologies) per manufacturer’s instructions.

### RNA-seq analysis

Bulk RNA-seq was performed by Novogene. A total of 6 samples were used for these experiments. To prepare these samples, total RNA from NK-92 cells treated with 10 µg/ml of UMCD6 or IgG for 6 h was extracted using RNAeasy MiniPrep Kit (Qiagen). Samples were then sequenced on an Illumina Hiseq platform and 125 bp/150 bp paired-end reads were generated. Index of the reference genome was built using Bowtie v2.2.3 and paired-end clean reads were aligned to the reference genome using TopHat v2.0.12. Differential gene expression analysis was performed using DESeq2. *P* values were adjusted using the Benjamini & Hochberg method. Corrected *P* value of 0.05 and log2 (Fold-change) of 1 were set as the threshold for significantly differential expression.

### RT-PCR analysis

NK-92 cells were treated with 10 µg/ml of UMCD6 or IgG and harvested at 6 h. RNA was extracted using Direct-zol RNA MiniPrep (Zymo Research). mRNA expression was measured using SYBR Green PCR Master Mix Reagent (Thermo Fisher Scientific) and the following primers: NKG2D F: 5′-TTCAACACGATGGCAAAAGC-3′, NKG2D R: 5′-CTACAGCGATGAAGCAGCAGA-3′, HCST F: 5'-TCTGGGTCACATCCTCTTCCT-3', HCST R: 5'-AAGTGCCAGGGTAAAAGGCAG-3′), CD244 F: CD244-F: 5’-ACAGGTTGCAAGGCAGTTCT-3′ and CD244 R: 5’- GTGAGGGCAGCAACTTCTTC-3’. Triplicates were performed for each sample. Real abundance for each gene was calculated using the ΔΔCT method and β-actin was used as an internal control.

### Western blotting

1 × 10^6^ NK-92 cells were first treated with UMCD6 or an IgG control antibody at 10 µg/ml and cell lysates were collected after 72 h. Samples containing 15 µg of protein were separated by Tris–Glycine SDS-PAGE (Sigma Aldrich) and electro-blotted onto nitrocellulose membranes. To measure changes in PI3K and mTOR expression, HRP-conjugated antibodies to PI3K (Cell Signaling, Cat#428 T), and mTOR (Cell Signaling, Cat#2972) were used at 1:1000 in 5% milk. β-actin (Cell Signaling, Cat#13E5) was used as a control for loading. Bands were imaged on an Amersham Imager 600RGB (GE Healthcare).

### Antibodies

UMCD6, a mouse anti-human monoclonal antibody that targets domain 1 of CD6, was created in our laboratory and was purified from mouse ascites and desalted by column chromatography using Protein G and dextran per manufacturer’s instructions (Thermo Fisher Scientific). Pembrolizumab and nivolumab (anti-PD-1) were obtained from Merck and Bristol-Myers Squibb. The following antibodies were used for flow cytometry analyses: FITC anti-human CD4 (BioLegend Cat#391503), PE anti-human CD45 (Biolegend, clone HI30), APC anti-human CD56 (Biolegend, clone 5.1H11), APC/Cyanine7 anti-human CD8a (Biolegend, San Diego, CA, USA, clone RPA-T8), PE/Cyanine7 anti-human CD3 (Biolegend, clone HIT3a), PerCP/Cyanine5.5 anti-human CD314 (NKG2D) (Biolegend, clone 1D11), FITC anti-human CD6 (Biolegend, clone BL-CD6), APC anti-human CD16 (Biolegend, clone 3G8), Pacific Blue anti-human/mouse Ki67 (Biolegend, clone 16A8), FITC anti-human/mouse Granzyme B (Biolegend, clone GB11) and APC anti-human Perforin (Biolegend, clone B-D48).

### Immunohistochemistry

Xenograft tumors derived from breast cancer MDA-MB-231 cells were embedded in optimal cutting temperature compound (Sakura Finetek) for cryosectioning at 8 µm. Antibodies against CD56 (Biolegend, clone 5.1H11) were incubated at 1:100 dilutions overnight. The following day, slides were incubated with goat anti-mouse IgG-Cy3 antibodies (Jackson ImmunoResearch) for 1 h at room temperature. Slides were then washed with PBS to remove residual antibody and samples were mounted using a DAPI solution containing Prolong™ Gold antifade and mounting medium (Invitrogen). Fluorescence images were taken using an Olympus BX51 microscope (Olympus America Inc.).

### Statistical analysis

The statistical analysis for all the experiments was performed using Graph Pad Prism software (GraphPad Prism). In vivo bioluminescence, tumor-infiltrating lymphocytes, RT-PCR and western blot data are shown as mean ± standard deviation. Student’s *t* test or ANOVA were used for group comparisons for normally distributed data. Log-rank test was used to assess the statistical significance in survival. *P* values < 0.05 were considered to be statistically significant.

## Results

### UMCD6 augments killing by human PBMC of breast cancer cells xenotransplanted into immunodeficient mice and increases survival of treated mice

**Fig. 1:**
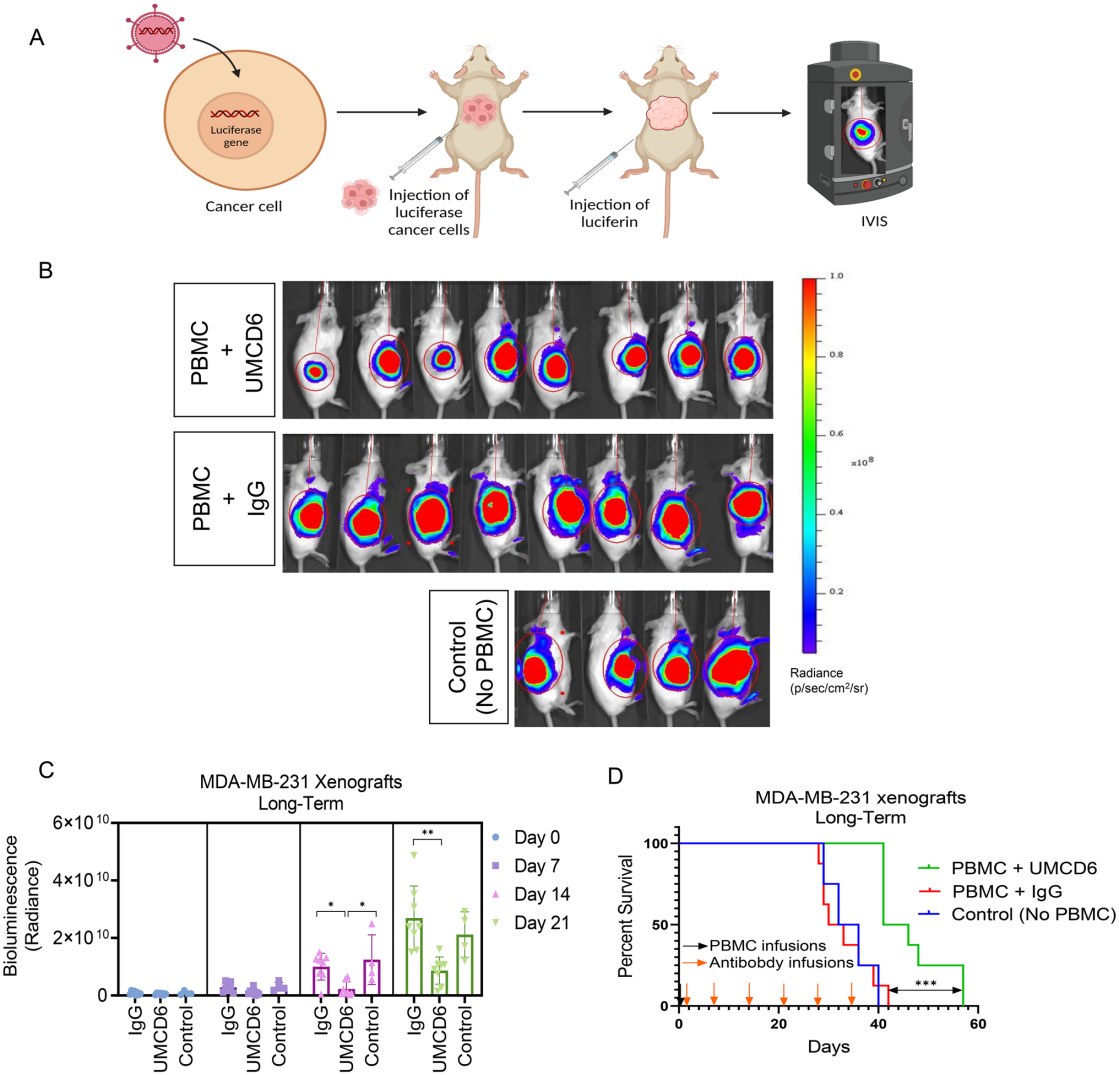
UMCD6 increases survival and augments killing by human PBMC of a breast cancer line xeno-transplanted into immunodeficient mice. A Schematic representation of in vivo visualization of tumor growth by the IVIS imaging system. B 2 × 10^6^ MDA-MB-231 cells were inoculated s.c. in the abdomen of female SCID/beige mice. Once tumors reached 1 cm, mice were administered 1 × 10^7^ human PBMCs by tail vein (day 0). The next day, mice were injected with 0.1 mg control IgG or UMCD6. IVIS images at day 14 are shown. C Tumor growth, measured by IVIS, showed a robust decrease in bioluminescence signal in mice treated with UMCD6 compared to IgG and control (not administered PBMCs nor antibodies). The effect of UMCD6 on tumor volume can be seen from day 14 after UMCD6 administration (*p < 0.05). Data represent mean of 4–8 animals ± SD. Group comparisons were done using 1-way ANOVA test at each specific timepoint. D Survival was significantly prolonged in the UMCD6 group compared to the IgG and control groups (***p < 0.001)

**Fig. 2:**
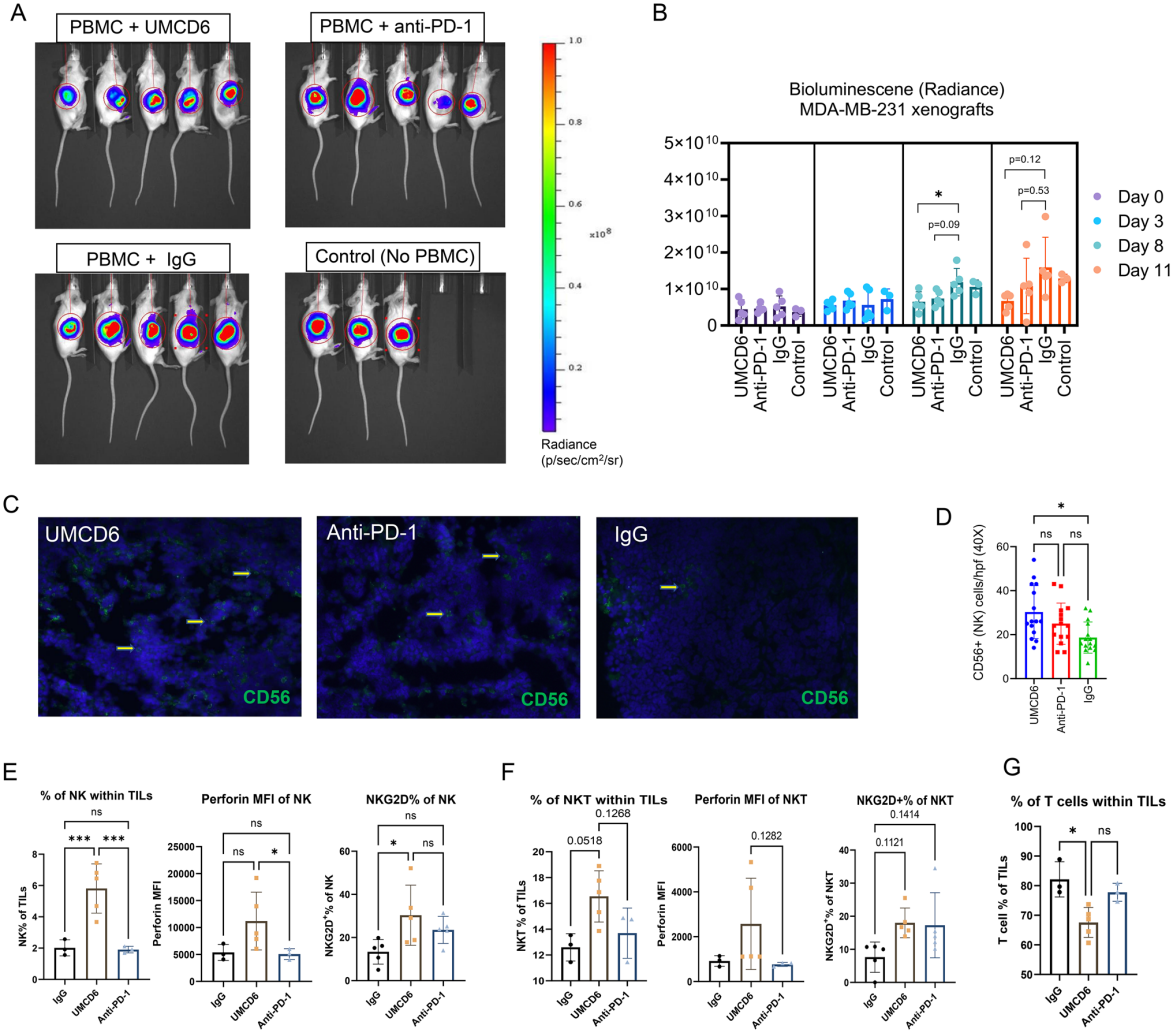
The effect of UMCD6 is more sustained than pembrolizumab in the killing of MDA-MB-231-derived xenograft tumors by PBMC. A and B We conducted a short-term in vivo experiment in which SCID/beige mice were first inoculated with MDA-MB-231 breast cancer cells, then administered 1 × 10^7^ human PBMCs and antibodies (UMCD6, pembrolizumab or IgG; 100 µg). Both UMCD6 and anti-PD-1 enhanced tumor killing by PBMC, but only UMCD6 showed statistical significance at day 8 (*p < 0.05). Data represent mean of 4–5 animals ± SD. C Representative pictures of tumor tissues immunostained for CD56 (human NK cell marker) at day 12 after treatment with UMCD6. D Immunofluorescence of tumor sections stained for the presence of NK cells showed that mice administered UMCD6 had an increased number of tumor-infiltrating NK cells compared to IgG control group (40X) (*p < 0.05). E Characterization of tumor-infiltrating lymphocytes (TILs) by flow cytometry shows that tumor-infiltrating NK cells are found in higher proportions in mice treated with UMCD6. NK cells are more abundant in UMCD6-treated mice (average of 5.8% among TILs) compared with IgG (2.01%) and anti-PD-1 (1.92%). Moreover, tumor-infiltrating NK cells from UMCD6-treated xenografts express higher levels of perforin (*p < 0.05 vs Anti-PD-1) and the activating receptor NKG2D (*p < 0.05 vs IgG). F Similarly, NKG2D and perforin expression in tumor-infiltrating NK-T cells is present after treatment with UMCD6, although the increase was non-significant. G Percentage of T cells within TILs

To investigate the long-term efficacy of UMCD6, we generated a xenograft mouse model of breast cancer by subcutaneous injections of 2 × 10^6^ luciferase-labeled MDA-MB-231 cells into SCID/beige mice. When tumors reached 1 cm in diameter, mice were infused with 1 × 10^7^ human PBMC and treated, beginning the day after the PBMC infusion, with 100 µg of UMCD6 or control IgG antibody once a week. Tumor volume, measured by bioluminescence signal (Fig. 1A), was significantly reduced by UMCD6 at day 14 and such effect was maintained until the end of the experiment (*p < 0.05) (Figs. 1B and C). Exit from the study was defined for each mouse as the day on which tumors reached ≥ 2 cm in diameter and/or ulceration covered ≥ 50% of the tumor region. A robust increase in survival can be seen in the UMCD6 treated mice (100% alive, *n* = 8) compared to IgG-treated (25% alive, *n* = 8) and control mice (25% alive; *n* = 4) at day 38 (Fig. 1D). In a parallel experiment in which the main goal was to compare the short-term efficacy of UMCD6 versus anti-PD-1 therapy in vivo, we found that the effect of UMCD6 might be more sustained in the killing of MDA-MB-231-derived xenograft tumors by PBMC than the effect of anti-PD-1. As seen in Fig. 2A, both UMCD6 and pembrolizumab enhanced tumor killing by PBMC compared to IgG control, but only UMCD6 showed statistical significance at day 8 (*p < 0.05) (Fig. 2B). Tumor weight, measured 12 days following treatment initiation, shows both UMCD6 and anti-PD-1 decreased tumor volume, but only UMCD6 was statistically significant (*p < 0.05) (Supplementary Fig. 1). Consistent with these findings is that NK cells (CD56 +) were found in higher proportions in histological sections from tumors from UMCD6-treated MDA-MB-231 xenografts in comparison with tumor sections from the IgG-treated group (*p < 0.05). Interestingly, the increase in the number of tumor-infiltrating NK cells with anti-PD-1 therapy was not significant when compared to IgG (Fig. 2C and D). Next, to demonstrate that the mechanism of action of UMCD6 differs from conventional immunotherapies, we isolated and characterized a portion of the tumor infiltrating lymphocytes (TILs) by flow cytometry. In agreement with our immunofluorescence results, we found an increase in the percentage of tumor-infiltrating NK cells in UMCD6-treated mice (5.8%) compared with IgG (2.01%; *p < 0.001) and anti-PD-1 (1.92%; (*p < 0.001) (Fig. 2E). Moreover, tumor-infiltrating NK cells from UMCD6-treated xenografts expressed higher levels of NKG2D and perforin than those from IgG and anti-PD-1 treated mice (*p < 0.05) (Fig. 2E). The statistically significant increase in NKG2D and perforin expression in NK cells and the up-regulation trend in NK-T cells (Fig. 2F), is consistent with our previous findings *in vitro* [7] and indicates UMCD6 activation of NK cells in vivo*.* On the other hand, the percentage of total tumor-infiltrating T cells within the total TILs was reduced in the UMCD6 treated mice in favor of NK cells and NK-T cells (Fig. 2G).

### UMCD6 increases cytotoxicity of tumor-infiltrating lymphocytes (TILs)

**Fig. 3:**
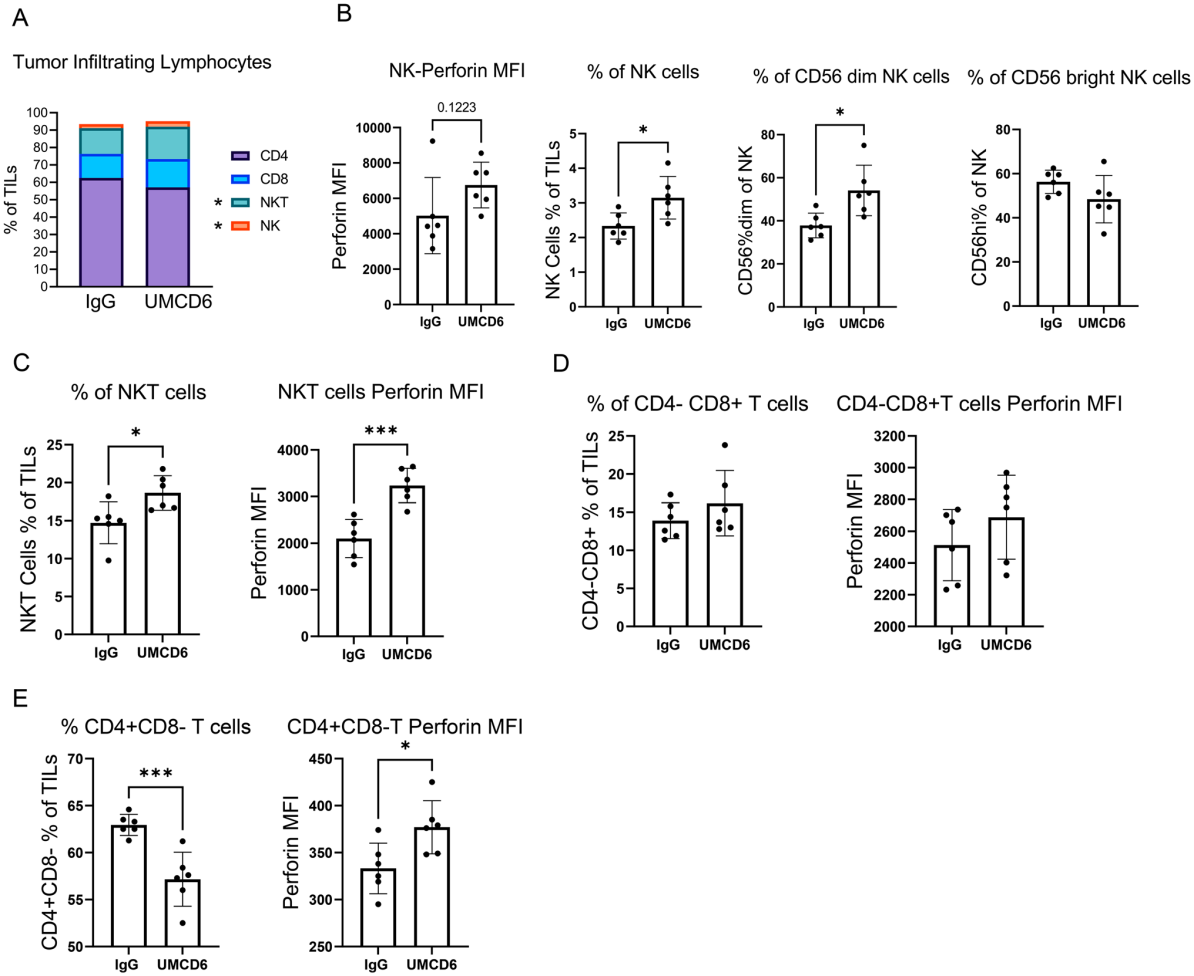
UMCD6 increases cytotoxicity of tumor-infiltrating lymphocytes (TILs). A TILs from breast cancer MDA-MB-231 xenograft tumors were analyzed by flow cytometry 4 days after treatment with UMCD6 or IgG antibodies. TILs from UMCD6-treated mice had higher levels of NK cells (3.14% ± 0.6), NKT cells (18.65% ± 2.25) and CD8 + T cells (16.18% ± 4.28) compared to TILs from IgG-treated mice: NK cells (2.33% ± 0.37), NKT cells (14.72% ± 2.74) and CD8 + T cells (13.90% ± 2.33). On the contrary, CD4 + T cells from UMCD6-treated mice were found in lower proportions (57.16% ± 5.79) compared to IgG-treated mice (62.15% ± 6.28). B We found a significant increased frequency of NK cells (*p < 0.05), specifically CD56 dim cells (*p < 0.05), in UMCD6-treated mice compared to IgG-treated mice. C CD3 + CD56 + NKT cells from UMCD6-treated mice were found to have enhanced perforin expression compared to control-treated mice (*p < 0.05). D CD8 + T cells were found in all mice and showed expression of perforin in UMCD6-treated mice (n.s. versus control group). E UMCD6 down-regulated the percentage of CD4 + T cells (****p < 0.001) while perforin production was significantly up-regulated. Data represent mean of 4–5 animals ± SD. An unpaired t-test was used to analyze differences between TILs from UMCD6 or IgG treated mice

We recently showed that UMCD6 binding to CD6 initiates CD6 capping and complete internalization from the cell surface of human lymphocytes within 6 h in vitro. When CD6 is internalized, changes in gene and protein expression of several activating and inhibitory receptors occur [7]. These changes were observed at the protein level between 48 and 72 h. Now, to study the phenotypic changes that occur on human lymphocytes upon treatment with UMCD6 in vivo, we collected and characterized TILs from our MDA-MB-231 xenografts 4 days post-treatment with anti-CD6, a time point when cells that had internalized CD6 are activated, but do not yet re-express CD6 on the cell surface. Frist, we confirmed that CD6 expression was robustly down-regulated on virtually all TILs (Supplementary Fig. 2), demonstrating that treatment with UMCD6 in vivo had effects on CD6 surface expression identical to what had previously been observed *in vitro* [7]. Importantly, TILs from UMCD6-treated mice showed an increased frequency of NK cells, (specifically CD56 dim cells which are known to be more cytotoxic than the CD56 bright cells), as well as NKT cells, when compared with TILs from IgG-treated mice (*p < 0.05). On average, TILs from UMCD6-treated mice comprised a mixture of NK cells (3.14% ± 0.6), NKT cells (18.65% ± 2.25), CD8 + T cells (16.18% ± 4.28) and CD4 + T cells (57.16% ± 5.79), whereas TILs from IgG-treated mice showed lower proportions of NK cells (2.33% ± 0.37), NKT cells (14.72% ± 2.74) and CD8 + T cells (13.90% ± 2.33), but higher proportions of CD4 + T cells (62.15% ± 6.28) (Fig. 3A, B and Supplementary Fig. 3). Perforin production was significantly up-regulated by NK-T cells and a small portion of CD4 + T cells (*p < 0.05) (Fig. 3C and E). CD8 + T cells from UMCD6-treated mice were found to express more perforin but the increase was not significant (Fig. 3D).

### UMCD6 modulates the expression of key receptors and granzyme genes in NK cells

**Fig. 4:**
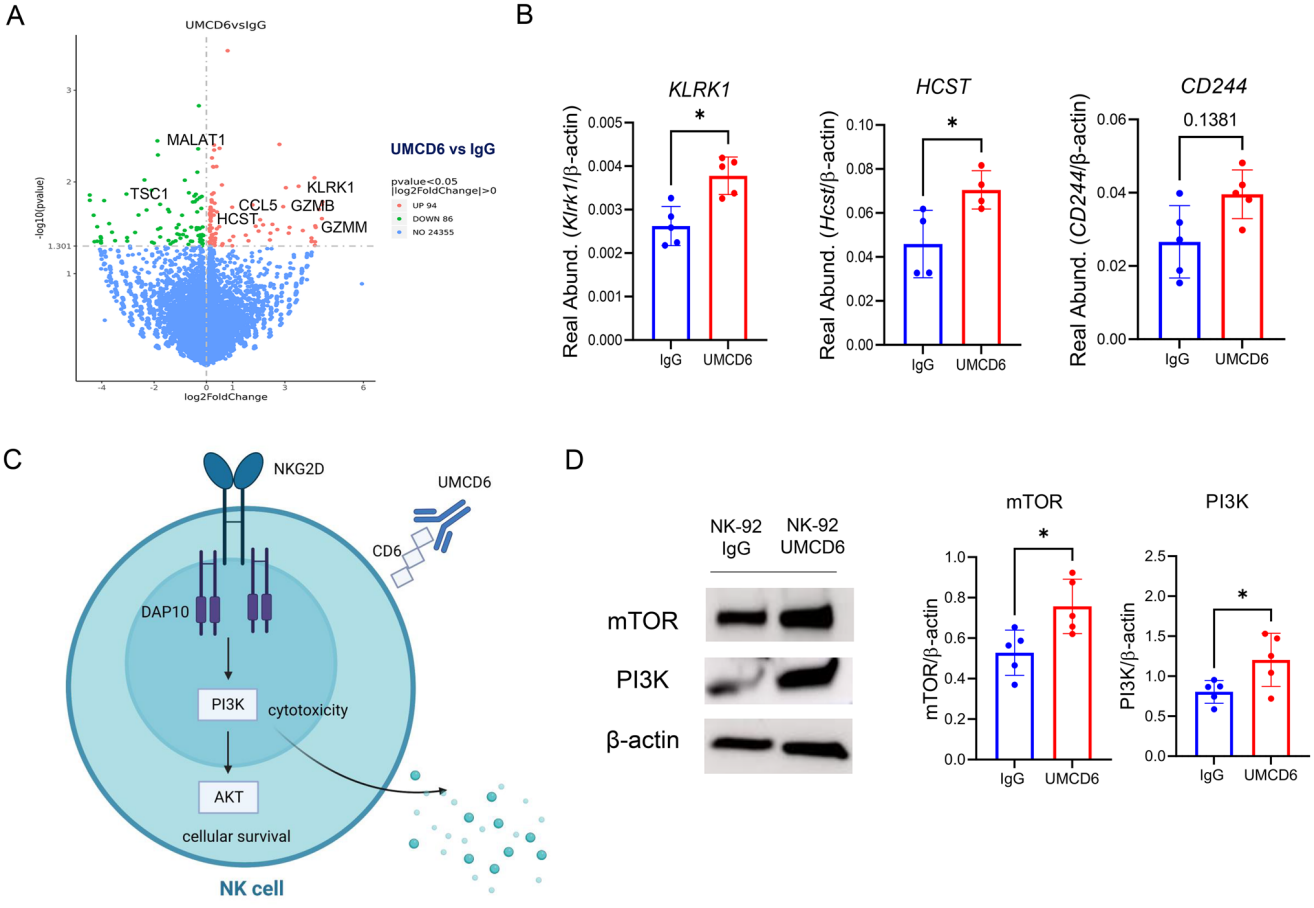
UMCD6 alters gene expression in human NK cells to enhance cytotoxic function. A RNA-seq data obtained using NK-92 cells stimulated with UMCD6 in a 6 h culture shows widespread changes in gene expression of 180 genes. B Up-regulation of the activating NK receptors NKG2D (*Klrk1*) and DAP10 (*Hcst*), shown to be involved in the activation of NK cells, was confirmed by RT-PCR. C Schematic representation of the role of UMCD6 in the activation of NK cells. Internalization of CD6 by UMCD6 up-regulates the expression of the activating receptor NKG2D-DAP10 and PI3K pathway. D PI3K and mTOR expression, down-stream pathways of NKG2D-DAP10 signaling complex, were found up-regulated at protein level in a 72 h co-culture of NK-92 cells with UMCD6. Data expressed as mean ± SD and p < 0.05 was considered significant (paired t-test)

We performed RNA-seq using the NK cell line NK-92 after culture with UMCD6 and compared the transcriptomic profile to that of IgG-treated NK-92 cells. Despite the highly activated status of NK-92 cells [13], our results showed widespread changes in gene expression induced by UMCD6 in a 6 h culture. 180 genes were altered significantly, with 94 of them being up-regulated and 86 down-regulated (Fig. 4A). These genes include several activating receptors and others that fall into categories such as granzyme production, activation of chemokines and genes whose protein products are viewed as important in the tumor microenvironment. Among the most important up-regulated genes, we found the potent activating receptor *KLRK1* [NKG2D] and its transmembrane signaling adaptor *HCST* [DAP10] (*p < 0.05), a receptor complex essential for optimal NK cell and CD8 + T cell activation [14]. *CD244* [2B4], another NK activating receptor, showed a trend towards up-regulation by UMCD6 (*p* < 0.1). Importantly, several granzyme-related genes were up-regulated. These include granzyme-M (*p < 0.05) and granzyme-B (*p < 0.05). Notably, *CCL5* (chemokine ligand 5), whose expression is associated with recruitment of NK cells and dendritic cells in the tumor microenvironment [15, 16], was robustly up-regulated upon treatment with UMCD6 (**p < 0.01). Among the most significant down-regulated genes, we found *TSC1* (TSC Complex Subunit 1), a negative regulator of NK proliferation [17], and *MALAT1* (Metastasis Associated Lung Adenocarcinoma Transcript 1), a negative regulator of Th1 and Th2 differentiation (**p < 0.01). Next, we validated the RNA*-*seq results performing qRT-PCR analyses and confirmed up-regulation of the NKG2D-DAP10 receptor complex on NK cells upon activation with UMCD6 (Fig. 4B). Recent literature has demonstrated that NKG2D-DAP10 ligation triggers cytotoxicity in human NK cells by activation of the phosphoinositide 3-kinase (PI3K) pathway [14, 18, 19]. Our data demonstrate that UMCD6 up-regulates NKG2D/DAP10 expression and activates the PI3K and mTOR pathways on human NK cells, suggesting that one mechanism of activation of NK cells by UMCD6 could be by activation of the NKG2D-DAP10 complex and its downstream pathways (Figs. 4C and D).

### UMCD6 enhances NK killing of human breast cancer cells in vivo

**Fig. 5:**
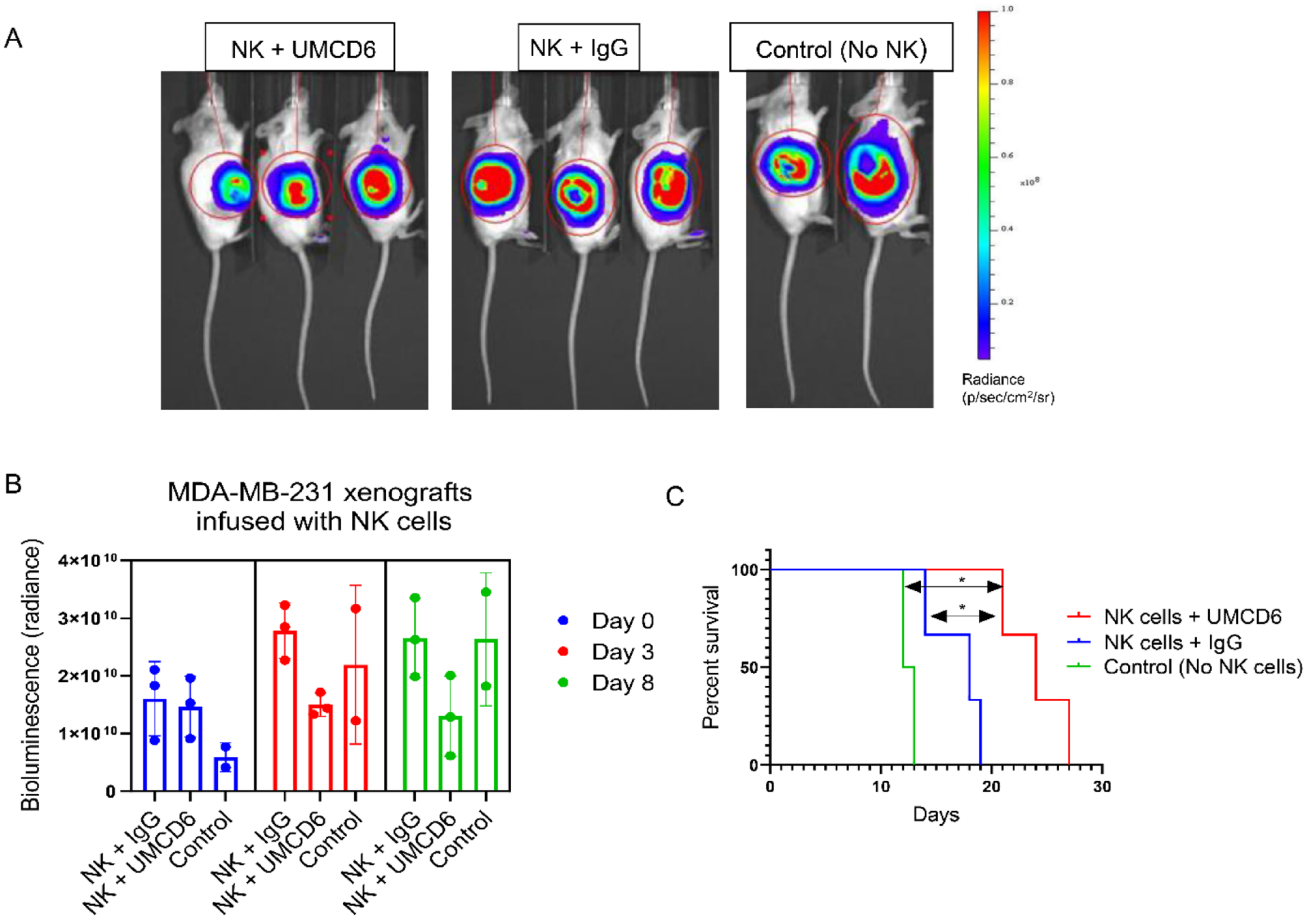
UMCD6 enhances NK killing of human breast cancer cells in vivo. A Bioluminescence images of breast cancer MDA-MB-231 SCID/beige xenografts infused with 1 × 10^6^ human NK cells and injected with 100 µg of antibodies (UMCD6 or IgG) at day 7. Control mice were not administered NK cells or antibodies. B Bioluminescence imaging of MDA-MB-231 mice revealed a decrease in tumor growth in mice receiving NK cells and UMCD6 at day 3 and 8 compared to mice receiving NK cells and IgG control antibody. Data represent mean of 2–3 animals ± SD. C Survival rate was significantly increased in the UMCD6 group compared to both the IgG and control groups. UMCD6 vs. IgG, *p = 0.0246; UMCD6 vs. untreated, *p = 0.0389

Because UMCD6 directly activates NK cells without the need for CD4 + T cells *in vitro* [7], we tested the efficacy of UMCD6 to enhance killing of xenografted breast cancer cells by using human NK cells in vivo. MDA-MB-231 xenografts were infused with 1 × 10^6^ human NK cells once (day 0), followed by one injection of 100 µg of UMCD6 or IgG (day 1). Bioluminescence imaging of MDA-MB-231 tumor-bearing mice (Fig. 5) revealed a decrease in tumor growth in mice receiving NK cells and UMCD6 compared to IgG control. Moreover, survival was significantly enhanced in the UMCD6 group compared either of the IgG and control groups (UMCD6 vs. IgG, *p = 0.0246; UMCD6 vs. untreated, *p = 0.0389). We conclude that NK cells are directly stimulated by UMCD6*, *in vitro and in vivo, to kill cancer cells with significantly enhanced efficiency.

### UMCD6 enhances killing of patient-derived lung cancer micro-organospheres (MOS)

**Fig. 6:**
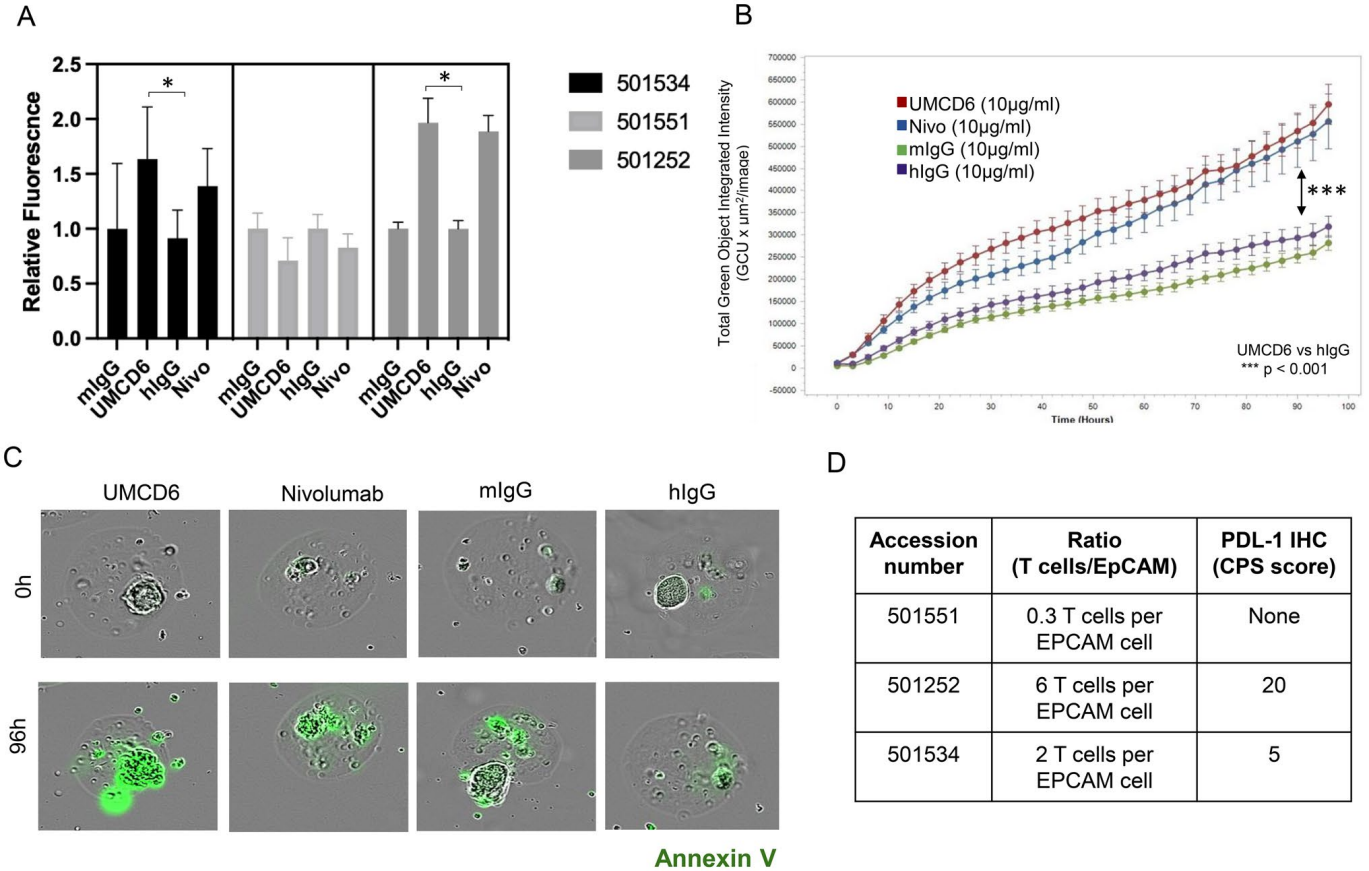
UMCD6 enhances killing of patient-derived lung cancer micro-organospheres (MOS). A Lung cancer MOS generated from cancer patient tissues were used in co-cultures with PBMC pre-incubated with UMCD6, nivolumab (anti-PD-1, nivo), mouse IgG or human IgG antibodies at 10 µg/ml. UMCD6 induces apoptosis of lung tumor cells in MOS, at least as efficiently as nivolumab, a PD-1 inhibitor in 2 out of 3 samples (501534 and 501252). Tumor cell killing was measured as the number and relative fluorescence of cancer cells in each well expressing Annexin-V (green fluorescence). B UMCD6 showed superiority to mouse and human IgG (after 12 h; (***p < 0.001)) and anti-PD-1 (at 42 h; (*p < 0.05); (data expressed as mean ± SD; green fluorescence, Annexin-V with y-axis linear, 1-way ANOVA). C Incucyte® images from lung cancer organoids (501252) at day 4 in the presence of antibodies. D T-cell/epithelial cell ratio (T cells/EpCAM ratio) and presence of PDL-1 were measured in each tumor MOS samples

To demonstrate that the anti-cancer effects of UMCD6 cannot be explained by allogeneic responses, we tested whether UMCD6 could enhance resident immune cell killing of patient-derived MOS. Lung cancer cells often over-express CD318 and UMCD6 has been shown to enhance killing of lung cancer cells by human PBMCs *in vitro* [7]. These experiments, in collaboration with Xilis, Inc., demonstrated that UMCD6 induced apoptosis of lung cancer cells, at least as efficiently as nivolumab (PD-1 inhibitor) in 2 out of 3 patients (Fig. 6A, B and C). The non-responder sample (501,551) had the lowest lymphocyte to epithelial cell ratio (T cells/EpCAM), which might explain why neither UMCD6 nor anti-PD1 induced lung cancer cell death in MOS from this patient (Fig. 6D).

### UMCD6 enhances prostate cancer killing by PBMC

We previously demonstrated that in in vitro co-culture models between prostate tumor cells and PBMC, UMCD6 augmented killing of LNCaP and PC3 prostate cancer cell lines through direct activation of NK and CD8 + T cells [7]. We now demonstrate that in vivo, UMCD6 increases survival and augments killing by human PBMC of PC3 cells xeno-transplanted into immunodeficient mice. The effect of UMCD6 on inhibiting tumor growth was measured by bioluminescence imaging and can be seen 10 and 14 days after UMCD6 administration. This effect was prolonged even though no additional infusions of lymphocytes were provided. Survival was significantly improved in the UMCD6 group compared to the pooled control group (**p < 0.01) (Supplementary Fig. 4).

### Pharmacodynamics of UMCD6

To gather information about the therapeutic potential of UMCD6, SCID/beige mice were infused with 1 × 10^7^ human lymphocytes and treated with one dose of UMCD6 or IgG control antibody (100 µg and 400 µg/mouse). Lymphocytes were recovered at day 1, 4, 7 and 14 from whole blood and expression/re-expression of CD6 on lymphocytes subsets was assessed by flow cytometry. As shown in (Supplementary Fig. 5), CD6 expression was reduced by at least 75% by day 4 in all cell subsets, and such effect was maintained until at least day 7. Complete re-expression of CD6 only occurred by day 14 on CD4 + NKT cells, but not on CD4 + or NK cells. An increase in CD6 expression on CD8 + NKT cells upon treatment with IgG suggests the existence of yet unknown mechanism that uniquely regulates CD6 expression by this subpopulation of lymphocytes.

## Discussion

The data presented in this report provide mechanistic insights regarding the effects of UMCD6 on the enhancement of NK cell and T cell responses against CD318 + cancers in vivo. We demonstrated that UMCD6 increases survival and augments killing by both PBMC and isolated NK cells of both triple-negative breast cancer and prostate cancer cells xenografted into immunodeficient mice. Of particular interest from both cancer immunotherapy and autoimmunity perspectives is the demonstrated ability of UMCD6 to prevent or reverse multiple models of human autoimmune disease [10], which provides an important advantage compared to current checkpoint inhibitors.

NK cells are increasingly becoming important components of therapeutic strategies for cancer because of their ability to kill tumor cells in a non-MHC-restricted manner. Our analysis of TILs confirmed that augmentation of lymphocyte cytotoxicity by UMCD6 is due to effects of this antibody primarily on NK cells, but also NKT cells, CD8 + T cells and even a small fraction of CD4 + T cells. Moreover, in vivo experiments using a single infusion of a small number of purified human NK cells, also increased survival of MDA-MB-231 xenografts.

Current understanding of the function of cytotoxic lymphocytes encompasses roles for many stimulatory and inhibitory receptors. In this report, we have demonstrated that augmentation of lymphocyte cytotoxic function by UMCD6 occurs when CD6 is internalized and no longer on the cell surface of NK cells. This functional change increases the expression of at least two activating receptors (NKG2D and 2B4) and their downstream signaling pathways (PI3K and mTOR), as well as various granzymes. Beyond the changes in gene expression observed thus far, it will be important to determine whether the anti-cancer effects of UMCD6 include the ability to revert expression of cell surface markers of CD8 T cell and NK cell exhaustion. Single cell RNA-seq and the use of humanized immune mice will be useful approaches to address these issues.

Natural killer-T cells (NKT cells) are a unique subset of CD1d-restricted T cells that share characteristics of both the innate and adaptive immune systems. As in NK cells, NKT cells rely on the balance between stimulatory and inhibitory signals via modulating the expression of several activating and inhibitory receptors [20]. In addition, NKT cells express CD16 which is known to trigger antibody-dependent cellular cytotoxicity by NK cells, making them an ideal target for the development of cancer immunotherapy. Our recent data demonstrate the importance of NKT cells in mediating killing of cancer cells in vivo upon activation with UMCD6, likely due to increase in the expression of NK-like activating receptors. Importantly, antibody-dependent cellular cytotoxicity is not an explanation for the effects of UMCD6, since this antibody does not bind to the cancer cells. Instead, binding and internalization of UMCD6 and cell surface CD6 directly alters the program of gene expression in lymphocytes, while controlling the effects of signals from CD6 ligands on cancer cells.

The ability of UMCD6 to enhance killing of patient-derived cancers in MOS demonstrates efficacy of UMCD6 in fully autologous human cancers ex vivo. This technology provides an excellent setting to understand and predict patient-specific responses because it uses organoids that retain the intratumoral heterogeneity and tumor clonal hierarchy from the patient’s own tumor. Our studies demonstrating that UCMD6 induces apoptosis of lung cancer cells in MOS, at least as efficiently as nivolumab, coupled with our previous data showing the ability of UMCD6 to suppress and control autoimmune diseases, fortify the pre-clinical rationale for the study of anti-CD6 as an effective and safer approach for cancer immunotherapy.

### Supplementary Information

Below is the link to the electronic supplementary material.

Supplementary file 1 (DOCX 1418 kb)
