# Supplementary material for: Activation of cytotoxic lymphocytes through CD6 enhances killing of cancer cells
Source: Cancer Immunol Immunother. 2024 Jan 27;73(2):34. doi: 10.1007/s00262-023-03578-1 (PMC10821976; doi:10.1007/s00262-023-03578-1)
Supplement: Supplementary file 1 — (DOCX 1418 kb) [file 262_2023_3578_MOESM1_ESM.docx]

**Supplementary Figure 1**

**Supplementary Figure 1. Tumor weight from MDA-MB-231 xenografts implanted in female SCID/beige mice infused with human PBMCs and treated with UMCD6, anti-PD-1 or IgG for 21 days.** UMCD6 decreased tumor volume compared to IgG and control (No PBMCs) (*p < 0.05). Data represents mean of 3-5 animals ± SD.

**Supplementary Figure 2**


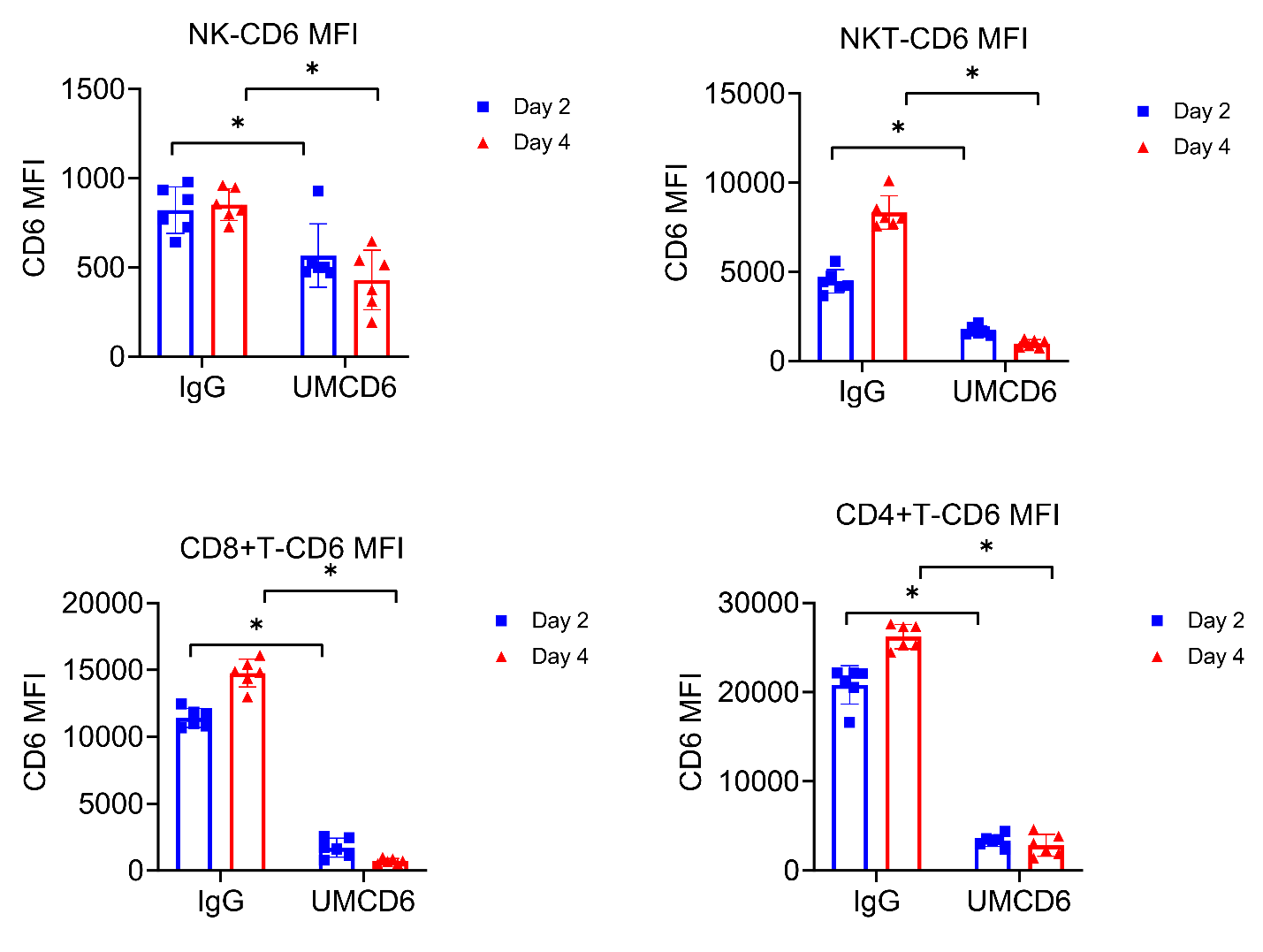


**Supplementary Figure 2. CD6 expression on Tumor-Infiltrating Lymphocytes from MDA-MB-231 xenografts treated with UMCD6.** CD6 expression was assessed by flow cytometry. We found a robust down-regulation on all lymphocyte subsets, suggesting that UMCD6 reduces CD6 cell surface expression *in vivo*. (**p* < 0.05 for UMCD6 vs IgG comparison).

**Supplementary Figure 3**


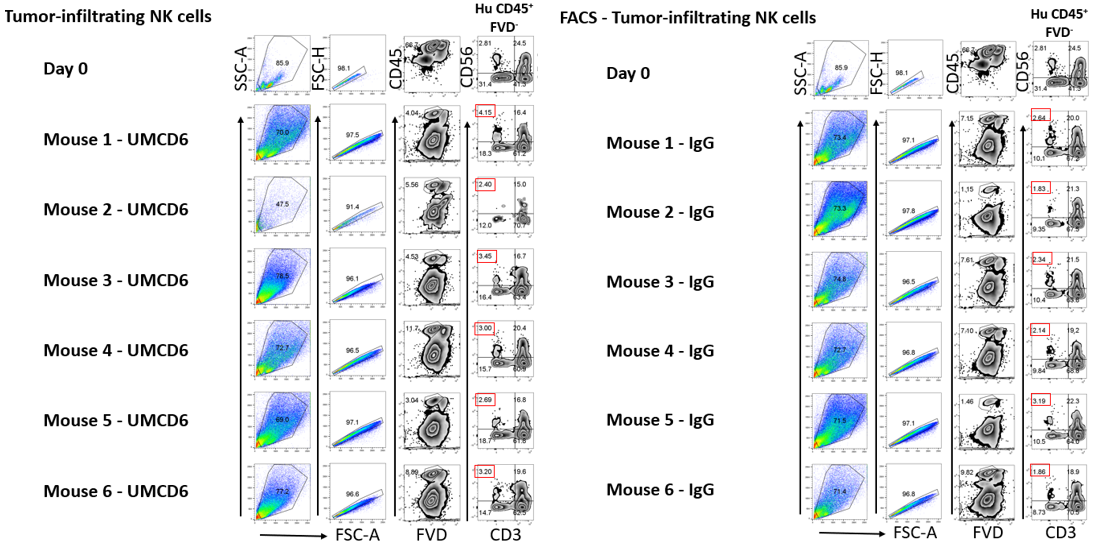
 **Supplementary Figure 3. Percentages of NK cells among the total human CD45+ cells in MDA-MB-231 xenografts infused with human PBMC.** Flow cytometry gating strategy for tumor-infiltrating NK cells isolated from MDA-MB-231 xenograft tumors. Left: Tumor-infiltrating NK cells were isolated and characterized from 6 MDA-MB-231 xenografted mice infused with human PBMCs and treated with UMCD6 for 4 days. Antibodies to CD45, CD56 and CD3 were used to determine the percentage of NK cells found in the tumors. Right: Similarly, tumor-infiltrating NK cells measured from 6 MDA-MB-231 xenografted mice infused with human PBMCs and treated with IgG.

**Supplementary Figure 4**


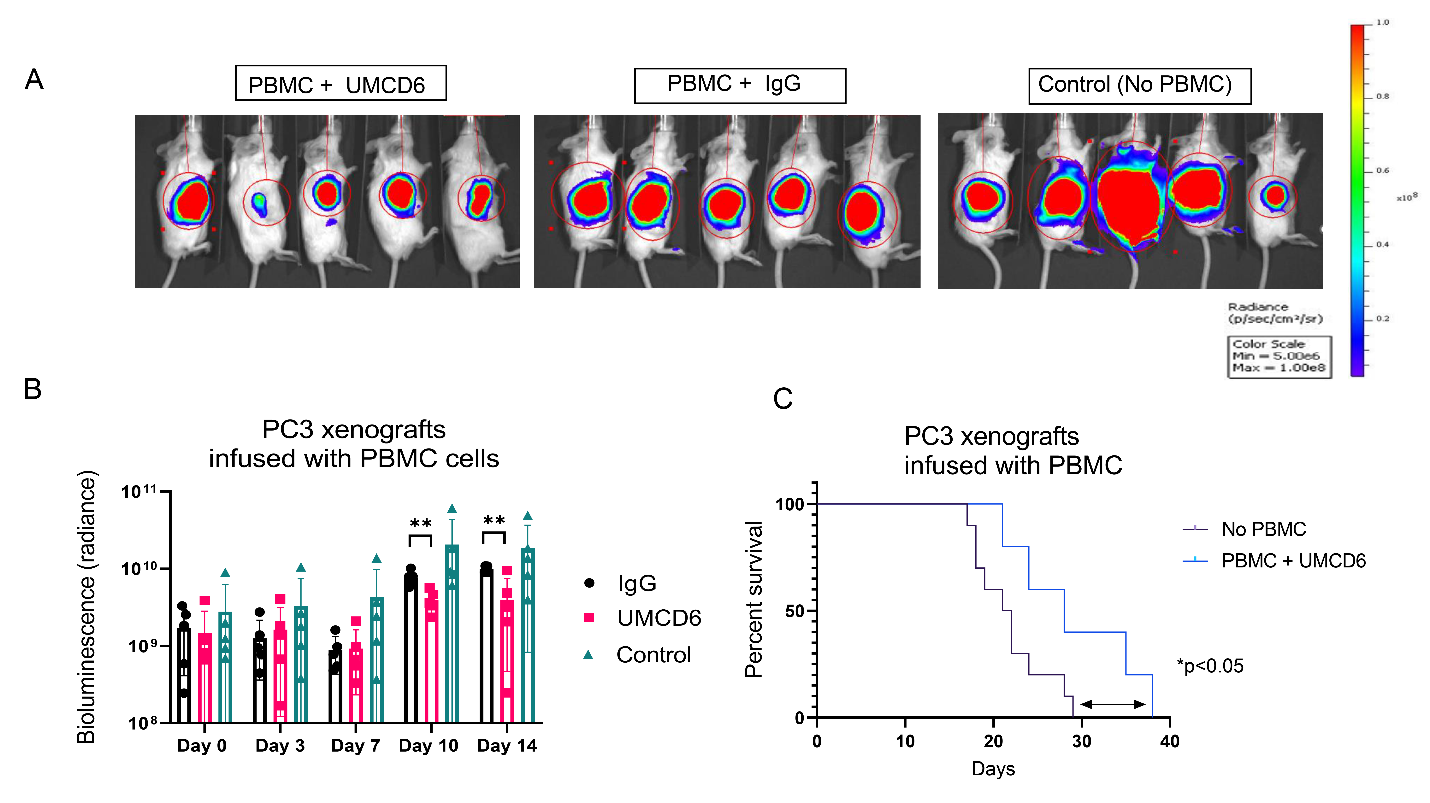


**Supplementary Figure 4. UMCD6 confers survival advantage in a human prostate cancer xenograft model.** A: SCID/beige mice were injected with the prostate cancer line PC-3 (2x10^6^ cells/mouse). At day 14, when prostate cancers were clearly growing by the In Vivo Imaging System, mice were infused with 1x10^7^ human PBMC. Mice that received PBMC also received weekly intraperitoneal injections of antibodies, UMCD6 or control IgG (100µg/mouse). The effect of UMCD6 on slowing tumor volume growth can be seen 14 days after administration with UMCD6. B: Bioluminescence imaging revealed a decrease in prostate cancer growth by PBMC and UMCD6 at day 10 and day 14 (**p* < 0.05). C: Survival was significantly prolonged in the UMCD6 group compared to the pooled control groups (**p* < 0.05).

**Supplementary Figure 5**


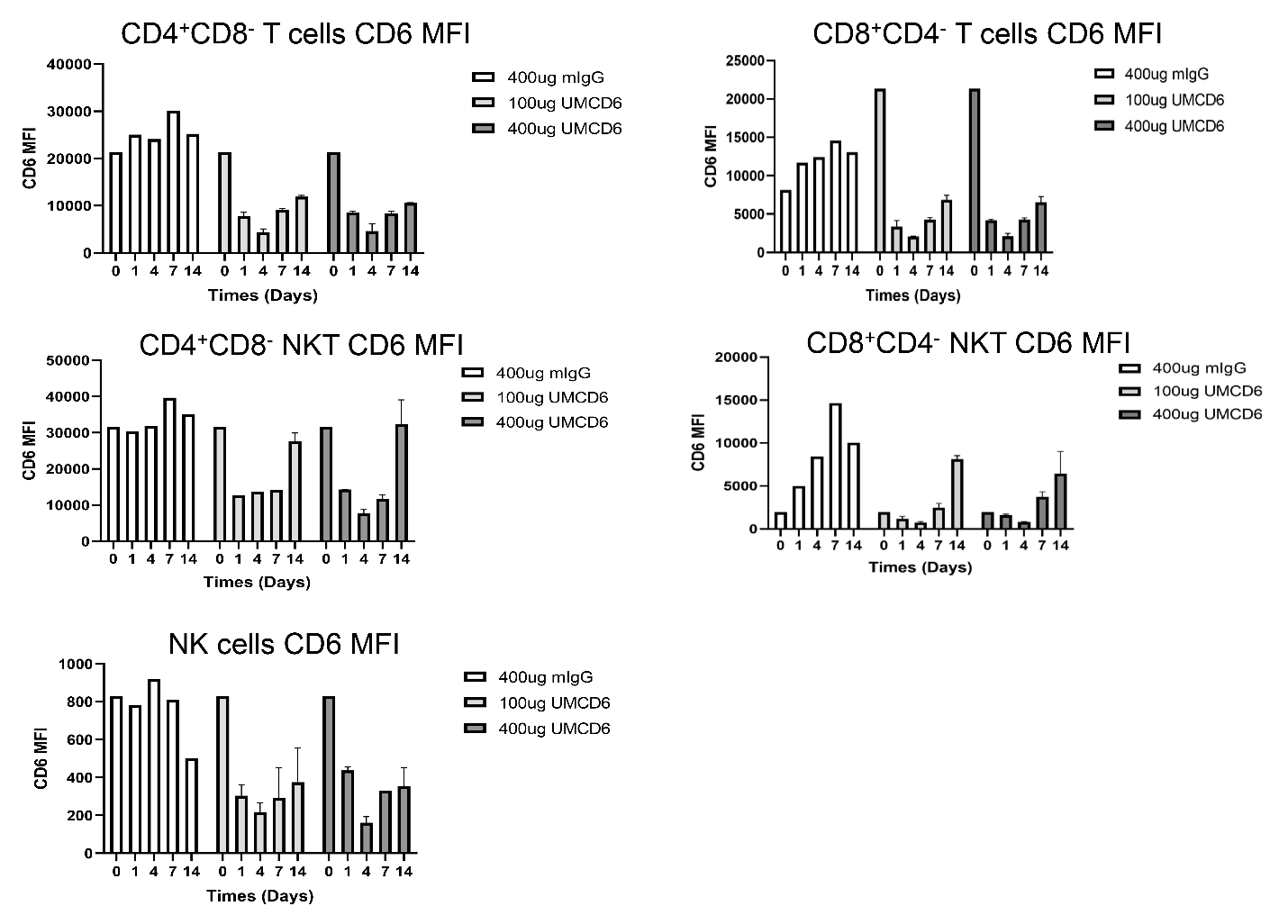


**Supplementary Figure 5. Pharmacodynamics of UMCD6.** Immunodeficient SCID/beige mice were infused with 1x10^7^human lymphocytes via tail vein and treated with 2 different concentrations of UMCD6 or IgG control antibody (100 µg and 400 µg/mouse). Human lymphocytes were recovered at day 1, 4, 7 and 14 from whole blood and cells were analyzed for their CD6 expression by flow cytometry. CD6 expression was robustly reduced by day 4 in CD4+, CD8+, NK and NKT cells, and such effect was maintained until at least day 7. Completely re-expression of CD6 only occurred by day 14 on CD4+ NKT cells, but not on other cell subsets.
